# Association of diet quality with dietary inflammatory potential in youth

**DOI:** 10.1080/16546628.2017.1328961

**Published:** 2017-06-07

**Authors:** Rowaedh Ahmed Bawaked, Helmut Schröder, Lourdes Ribas-Barba, Maria Izquierdo-Pulido, Carmen Pérez-Rodrigo, Montserrat Fíto, Lluis Serra-Majem

**Affiliations:** ^a^ Cardiovascular Risk and Nutrition Research Group (CARIN), IMIM (Hospital del Mar Medical Research Institute), Barcelona, Spain; ^b^ Department of Experimental and Health Sciences, University of Pompeu Fabra, Barcelona, Spain; ^c^ CIBER Epidemiology and Public Health (CIBERESP), Instituto de Salud Carlos III, Madrid, Spain​​​​​; ^d^ Fundación para la Investigación Nutricional (Nutrition Research Foundation), Barcelona, Spain​​​​​; ^e^ CIBER Physiopathology of Obesity and Nutrition (CIBEROBN), Instituto de Salud Carlos III, Madrid, Spain​​​​​; ^f^ Department of Nutrition, Food Sciences and Gastronomy, University of Barcelona, Barcelona, Spain; ^g^ FIDEC Foundation, University of the Basque Country (UPV/EHU)Bilbao, Bilbao, Spain; ^h^ Research Institute of Biomedical and Health Sciences, University of Las Palmas de Gran Canaria, Las Palmas, Spain​​​​​

**Keywords:** Dietary inflammatory index, KIDMED, energy density, total dietary antioxidant, children, adolescents, enKid

## Abstract

**Background**: Diet plays a crucial role in the regulation of chronic inflammation. The sparse evidence available in adult populations indicates that diet quality is linked to the dietary inflammatory potential; however, this association has not been established in youth.

**Design**: Data were obtained from a representative national sample of 2889 children and young people in Spain, aged 6–24 years. The dietary inflammatory potential was measured by the dietary inflammatory index (DII), and diet quality by three conceptually different measures: the Mediterranean Diet Quality Index for children and adolescents (KIDMED), energy density, and total dietary antioxidants capacity.

**Results**: The mean DII was 1.96 ± 0.76 units Scoring for the KIDMED index and the total dietary antioxidant capacity significantly decreased (*p* < 0.001 and *p* = 0.030, respectively) across quintiles of the DII, whereas the opposite was true for energy density (*p* < 0.001). The effect size of these associations was strongest for energy density, followed by the KIDMED index and total dietary antioxidant capacity.

**Conclusion**: A healthy diet characterized by high adherence to the Mediterranean diet, high total dietary antioxidant capacity, or low energy density was linked to greater anti-inflammatory potential of the diet, as measured by the DII.

## Introduction

Diet plays a crucial role in the regulation of chronic inflammation [1,2]. The Western dietary pattern, high in refined grains, sugars, simple carbohydrates, red meat, and high-fat dairy products, increases the levels of pro-inflammatory markers such as C-reactive protein (CRP) and interleukin-6 (IL-6) [1,3]. In contrast, a traditional Mediterranean diet with generous consumption of fruits, vegetables, whole grains, legumes, increased consumption of fish and nuts, and higher use of olive oil in food preparation is associated with lower levels of pro-inflammatory biomarkers, including endothelial adhesion molecules, CRP, and tumor necrosis-α (TNF-α) [2]. Evidence shows that several chronic diseases, including cardiovascular diseases and cancer, are triggered in part by inflammation [4,5], and atherosclerosis progresses from childhood and adolescence to adulthood [6].

A high score on the dietary inflammatory index (DII), which was developed to assess the inflammatory potential of the diet [7], has been associated with inflammation biomarkers such as CRP [8,9], IL-6 [10,11], homocysteine [10], and TNF-α [11]. In addition, an increased DII score has been associated with cancer [12], asthma [13], and cardiovascular diseases [14].

Limited evidence has positively associated diet quality with the dietary anti-inflammatory potential in adult populations [15–17], but data for children and young people are missing. Furthermore, there is no consensus about the definition of diet quality [18]. Indeed, scientists have proposed many different measures of diet quality [18]. In this study, we hypothesized that high anti-inflammatory potential is characteristic of three conceptually different measures of high diet quality in youth: adherence to the Mediterranean diet, total dietary antioxidant potential, and energy density.

The objective of this study was to determine dietary inflammatory potential, measured by the DII, and its association with diet quality indicators in a representative sample of Spanish youth.

## Methods

### Study design

The enKid Study was a cross-sectional survey of the nutritional status and food habits of 3534 Spanish children and young adults, conducted between 1998 and 2000. Participants were selected by multistage random sampling procedures based on a population register. The objective of the enKid Study, described in detail elsewhere [19], was two-fold: (I) to establish the prevalence of micronutrient deficiencies in the population aged 2–24 years; and (II) to analyze the association of these micronutrients with gender and age groups.

The sample size was calculated according to (i) the estimated prevalence of most micronutrients with 95% confidence interval and an accuracy of ±2.5% of the average value of the micronutrient; and (ii) a statistical power of 80% to detect significant differences between two groups = 10% of the mean of the micronutrients (setting the alpha error at *p* = 0.05). The calculated sample size of 3850 individuals was overestimated by 30%, resulting in a theoretical sample size of 5500 individuals. The final sample size of the enKid Study was 3534 individuals (Supplementary Figure 1). We excluded 385 children aged 2–5 years to concentrate the study population in a narrower age range. Individuals with incomplete dietary data were also excluded (*n* = 250). The final sample consisted of 2889 individuals aged 6–24 years.

Parental written consent was obtained on behalf of each participant younger than 18 years. The study protocol was approved by the ethics committee of the Spanish Society of Community Nutrition.

### Dietary data collection

Dietary data were collected during in-home interviews carried out by 43 trained dietitians and nutritionists using household measures to estimate portion sizes. Dietary intake was assessed by means of a 24 h recall. A second 24 h recall was completed in a random sample of 25% of the participants on an independent non-consecutive day. The administration of the second questionnaire allowed for the adjustment of intakes for random intra-individual variation using the method described by Liu et al. [20]. The same field staff entered survey data into software specifically designed for the study.

### Dietary inflammatory index (DII)

The inflammatory properties of each participant’s diet were assessed from the 24 h recall data using the DII, which is based on a review and analysis of 1939 scientific articles [7]. These articles studied the relationship between 45 dietary components and six inflammatory markers (CRP, IL-1β, IL-4, IL-6, IL-10, and TNF-α) derived from cell culture and animal experiments, and from cross-sectional, longitudinal, and intervention trials in humans. Each food parameter in each article was scored by assigning (+1) for pro-inflammatory effect, (−1) for anti-inflammatory effect, or (0) for no effect, and weighted according to the study design. In the present study, 23 of the 45 DII food parameters were available (fiber, protein, carbohydrates, cholesterol, total fat, trans fat, saturated fat, monounsaturated fat, polyunsaturated fatty acids, omega-3 and omega-6 fatty acids, iron, magnesium, energy intake, and vitamins A, B_1_, B_2_, B_5_, B_6_, B_12_, C, D, and E).​​​​​ For each participant, each food parameter intake score was subtracted from the mean of 11 countries from around the world and divided by its standard deviation. *Z* scores and centered percentiles were calculated to reduce the effect of right skewing. For each food parameter, the centered percentile was multiplied by the overall inflammatory effect. All DII scores for the food parameters were then summed to create the overall DII score for each participant, which ranged from −6.77, representing maximum anti-inflammatory properties, to 7.79, representing maximum pro-inflammatory properties. The development and validation of the DII have been explained in greater detail elsewhere [7].

### Diet quality measures

Energy density and total dietary antioxidant capacity were calculated from the 24 h recalls. The Mediterranean Diet Quality Index for children and adolescents (KIDMED) index was based on a 16-item questionnaire administered separately from the recalls as part of the enKid Study [21,22].

#### Energy density

There is no consensus about the best method to measure dietary energy density [23]. To allow comparability with other studies, we present data based on dietary density calculations that included food only [energy intake (kcal) from all foods consumed divided by the corresponding weight (g) of the foods] and all foods together with all caloric beverages [energy intake (kcal) from all foods and caloric beverages consumed divided by the corresponding weight (g) of the foods and beverages].

#### Total dietary antioxidant capacity

Dietary total antioxidant capacity was estimated using published data of antioxidant capacity in foods measured by ferric-reducing antioxidant power (FRAP) assays [24].

#### KIDMED index

The KIDMED index estimates adherence to the Mediterranean diet in children and young adults, considering the principles that sustain Mediterranean dietary patterns and those that undermine it. Items denoting lower adherence were assigned a value of −1 (four items) and those related to higher adherence were scored +1 (12 items). Scores ranged from −4 to 12, with higher scores indicating greater adherence to the Mediterranean diet and scores below 6 points defined as low Mediterranean diet adherence.

### Covariates

Data on physical activity and maternal education were reported by participants responding to an interviewer-administered questionnaire. The physical activity questionnaire included 14 questions on type and frequency of physical activity and time spent at school and outside school doing physical activity. Maternal education level was recorded as: (i) no education (never went to school); (ii) primary education not completed; (iii) completed primary education; (iv) secondary education; and (v) university. Basal metabolic rate (BMR) was estimated with Schofield equations based on gender, age, weight, and height [25]. Implausible reporters of energy intake were identified by replacing Goldberg’s single cut-off [26] with age- and gender-specific cut-off values. These values consisted of the 95% confidence limits of agreement between physical activity level (PAL) and the ratio of energy intake to BMR. The following formula was used:

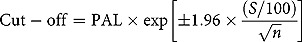

where

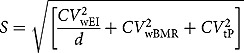

Intra-individual variations in energy intake (CV^2^wEI) and BMR (CV^2^wBMR) and inter-individual variation in PAL (CV^2^wtP) were calculated using gender- and age-specific reference values [27–29]. The single Goldberg PAL of 1.55 was replaced by gender- and age-dependent PAL. We estimated dietary intake by one 24 h recall and set the number of days to 1.

### Statistical analysis

General linear modeling procedures were used to compare baseline participant characteristics by quintiles of DII. Polynomial contrast was used to determine overall *p* for linear trend for continuous variables with normal distribution, and the Kruskal–Wallis test to determine overall *p* for non-normal distributions. The *p* for linear trend for categorical variables was obtained by the Mantel–Haenszel linear-by-linear association chi-square test.

To determine the association between DII and diet quality, we fitted general linear models adjusted for gender, age, energy intake, energy underreporting, region, community size, and maternal education level. *Z* scores of energy density, total dietary antioxidant capacity, and the KIDMED index were created to compare the effect size of the associations with the DII.

Calculation of Cohen’s *κ* was based on the observed versus the expected agreement and used to test the strength of agreement between the tertile distribution of energy density, total dietary antioxidant capacity, and the KIDMED index. To explore effect modification due to DII and energy overreporting and underreporting, we modeled interaction terms for DII/energy overreporting and DII/energy underreporting. Associations were considered significant if *p* < 0.05. SPSS for Windows version 18 (SPSS, Chicago, IL, USA) was used for all statistical analyses.

## Results

The mean DII was 1.25 ± 1.39 units, with a range of −4.27 to 4.16 (Table 1). Participants with high DII scores were more likely to be female, were younger, and had fewer total physical activity minutes per day. The proportion of participants with a high DII was greater in communities of 10,000–50,000 inhabitants and lower in communities of more than 350,000 inhabitants (Table 1).

**Table 1.** General characteristics across quintiles (Q1–Q5) of the dietary inflammatory index (DII).

	Q1 (*n* = 578)	Q2 (*n* = 579)	Q3 (*n* = 577)	Q4 (*n* = 578)	Q5 (*n* = 577)	***p***^a^
DII	0.79 (−1.17; 1.35)	1.63 (1.35; 1.87)	2.06 (1.87; 2.26)	2.42 (2.26; 2.61)	2.87 (2.61; 3.66)	<0.001
Male, *n* (%)	336 (58.1)	291 (50.3)	242 (41.9)	229 (39.6)	220 (38.1)	<0.001
Age (years)	19.2 (18.8, 19.6)	17.5 (17.1, 17.9)	16.9 (16.5, 17.3)	15.7 (15.3, 16.1)	14.5 (14.1, 14.9)	<0.001
Physical activity (min/day)	145 (134, 156)	134 (123, 145)	127 (116, 138)	130 (119, 141)	127 (116, 138)	0.024
Maternal education, *n* (%)^b^	112 (19.6)	99 (17.4)	93 (16.3)	108 (18.8)	87 (15.2)	0.460
Community size, *n* (%)^c^						<0.001
<10,000	130 (22.5)	119 (20.6)	132 (22.9)	126 (21.8)	126 (21.8)	
10,000–50,000	104 (18.0)	130 (22.5)	140 (24.3)	168 (29.1)	177 (30.7)	
50,000–350,000	150 (26.0)	158 (27.3)	150 (26.0)	175 (30.3)	163 (28.2)	
>350,000	194 (33.6)	172 (29.7)	155 (26.9)	109 (18.9)	111 (19.2)	
Region, *n* (%)^d^						0.837
Central	144 (24.9)	146 (25.2)	141 (24.4)	155 (26.8)	133 (23.1)	
Northeast	151 (26.1)	137 (23.7)	134 (23.2.6)	140 (24.2)	130 (22.5)	
North	120 (20.8)	112 (19.3)	142 (24.6)	128 (22.1)	142 (24.6)	
South	75 (13.0)	93 (16.1)	76 (13.2)	77 (13.3)	83 (14.4)	
East	71 (12.3)	73 (12.6)	64 (11.1)	63 (10.9)	70 (12.1)	
Canary Islands	17 (2.94)	18 (3.11)	20 (3.47)	15 (2.6)	19 (3.29)	

Data are shown as mean and range for DII; mean and 95% confidence interval for continuous variables (age and physical activity); and proportions within quintiles for categorical variables (male gender, maternal education level, community size, and region).

^a^*p* Values were obtained by ANOVA, Kruskal–Wallis, and Pearson chi-square for normal continuous, non-normal continuous, and categorical variables, respectively.

^b^Maternal education expressed as proportion with university degree.

^c^Percentage expressed as proportion within community size.

^d^Percentage expressed as proportion within region.

Adherence to the Mediterranean diet, total dietary antioxidant capacity, intake of protein, polyunsaturated fatty acids, fiber, magnesium, vitamins C, E, B_6_, B_2_, and B_1_, and intake of fruits, vegetables, legumes, and fish decreased across the DII. The opposite was true for dietary energy density and intake of lipids, monounsaturated fat, saturated fat, calcium, dairy, cereals, meat, pastry, and cakes and sweets. Higher DII was associated with a decreased proportion of energy underreporting and increased overreporting (Table 2).

**Table 2.** Diet quality across quintiles (Q1–Q5) of the dietary inflammatory index (DII).

Characteristic	Q1 (n = 578)	Q2 (n = 579)	Q3 (n = 577)	Q4 (n = 578)	Q5 (n = 577)	***p***^a^
KIDMED index	7.7 (7.5, 7.8)	7.2 (7.1, 7.4)	7.0 (6.8, 7.1)	6.9 (6.7, 7.1)	6.6 (6.5, 6.8)	<0.001
Dietary antioxidant capacity	11.4 (10.7, 12.1)	9.8 (9.0, 10.5)	9.0 (8.2, 9.7)	8.7 (7.9, 9.4)	7.6 (6.9, 8.4)	<0.001
Dietary energy density	0.93 (0.91, 0.96)	1.02 (0.99, 1.04)	1.06 (1.03, 1.09)	1.12 (1.09, 1.15)	1.2 (1.18, 1.23)	<0.001
Energy intake (kcal/day)	2272 (2239, 2304)	2073 (2040, 2105)	1974 (1941, 2007)	1927 (1895, 1960)	1951 (1919, 1984)	<0.001
Energy underreporting, *n* (%)	97 (16.8)	118 (20.4)	113 (19.7)	114 (19.7)	81 (14.1)	0.030
Energy overreporting, *n* (%)	19 (3.30)	5.0 (0.87)	3.0 (0.52)	4 (0.69)	7 (1.22)	<0.001
*E*% protein	18.0 (17.6, 18.4)	17.9 (17.5, 18.3)	18.2 (17.8, 18.6)	17.0 (16.6, 17.4)	16.1 (15.7, 16.5)	<0.001
*E*% lipids	39.0 (38.3, 39.7)	38.5 (37.8, 39.2)	38.6 (37.9, 39.3)	38.3 (37.6, 39.0)	41.0 (40.3, 41.7)	0.001
*E*% carbohydrate	44.8 (44.0, 45.6)	45.7 (45.0, 46.5)	44.9 (44.2, 45.7)	46.4 (45.6, 47.2)	45.1 (44.3, 45.9)	0.336
*E*% SFA	11.6 (11.3, 11.9)	12.3 (11.9, 12.6)	12.6 (12.3, 13.0)	13.2 (12.9, 13.5)	15.4 (15.1, 15.7)	<0.001
*E*% MUFA	15.9 (15.5, 16.3)	15.7 (15.3, 16.1)	16.1 (15.7, 16.4)	15.9 (15.5, 16.3)	16.8 (16.4, 17.1)	<0.001
*E*% PUFA	7.0 (6.8, 7.2)	6.0 (5.8, 6.2)	5.4 (5.2, 5.6)	4.8 (4.6, 5.0)	4.3 (4.1, 4.5)	<0.001
Fiber (g/1000 kcal)	8.5 (8.4, 8.6)	8.0 (7.9, 8.1)	7.7 (7.6, 7.8)	7.5 (7.4, 7.6)	6.9 (6.8, 7.0)	<0.001
Calcium (mg/1000 kcal)	425 (418, 431)	442 (436, 449)	442 (435, 449)	446 (439, 453)	444 (437, 451)	0.37
Magnesium (mg/1000 kcal)	141 (140, 143)	138 (137, 140)	137 (135, 138)	135 (133, 136)	127 (126, 129)	<0.001
Vitamin C (mg/1000 kcal)	47.9 (46.5, 49.2)	42.6 (41.2, 44.0)	39.8 (38.4, 41.1)	36.3 (35.0, 37.7)	27.7 (26.3, 29.1)	<0.001
Vitamin E (mg/1000 kcal)	3.9 (3.9, 4.0)	3.7 (3.6, 3.7)	3.4 (3.3, 3.5)	3.2 (3.1, 3.2)	2.9 (2.9, 3.0)	<0.001
Vitamin B_6_ (mg/1000 kcal)	0.87 (0.86, 0.88)	0.87 (0.85, 0.88)	0.86 (0.85, 0.87)	0.81 (0.80, 0.82)	0.75 (0.74, 0.76)	<0.001
Vitamin B_2_ (mg/1000 kcal)	0.81 (0.80, 0.83)	0.84 (0.82, 0.85)	0.84 (0.82, 0.85)	0.83 (0.82, 0.84)	0.82 (0.80, 0.83)	<0.001
Vitamin B_1_ (mg/1000 kcal)	0.67 (0.66, 0.68)	0.68 (0.67, 0.68)	0.67 (0.66, 0.68)	0.67 (0.66, 0.68)	0.62 (0.62, 0.63)	<0.001
**Food**						
Fruits (g/1000 kcal)	140 (132, 148)	109 (101, 117)	94.0 (86.7, 102)	76.6 (68.6, 84.6)	41.3 (33.3, 49.3)	<0.001
Vegetables (g/1000 kcal)	87.6 (82.4, 92.8)	61.2 (56.0, 66.4)	48.8 (43.6, 53.9)	36.9 (31.8, 42.1)	19.2 (14.0, 24.4)	<0.001
Dairy (g/1000 kcal)	158 (149, 168)	183 (173, 192)	190 (181, 200)	202 (193, 212)	213 (204, 234)	<0.001
Cereals (g/1000 kcal)	94.2 (90.5, 98.0)	92.9 (89.1, 96.7)	88.1 (84.3, 91.9)	91.1 (87.3, 94.9)	89.8 (86.0, 93.6)	<0.001
Meat (g/1000 kcal)	32.4 (28.9, 35.9)	32.2 (28.7, 35.8)	38.8 (35.3, 42.3)	37.3 (33.8, 40.8)	39.8 (36.2, 43.3)	<0.001
Legumes (g/1000 kcal)	12.8 (10.8, 14.8)	12.6 (10.6, 14.5)	10.5 (8.5, 12.4)	9.2 (7.2, 11.1)	5.7 (3.7, 7.7)	<0.001
Fish (g/1000 kcal)	44.6 (41.5, 47.8)	29.8 (26.7, 32.9)	24.3 (21.1, 27.4)	15.2 (12.1, 18.3)	8.68 (5.54, 11.8)	<0.001
Pastry (g/1000 kcal)	23.2 (20.6, 25.8)	20.7 (18.1, 23.3)	21.1 (18.5, 23.7)	23.9 (21.3, 26.5)	28.0 (25.4, 30.6)	0.002
Cakes, sweets (g/1000 kcal)	10.2 (9.05, 11.4)	11.3 (10.2, 12.5)	11.8 (10.6, 12.9)	13.3 (12.1, 14.5)	16.6 (15.4, 17.8)	<0.001

Data are shown as means and 95% confidence interval for continuous variables (KIDMED index score, dietary antioxidant capacity, dietary energy density, energy intake, nutrients, and foods), and proportions for categorical variables (energy underreporting and overreporting).

^a^*p* Values were obtained by ANOVA, Kruskal–Wallis, and Pearson chi-square for normal continuous, non-normal continuous, and categorical variables, respectively.

SFA, saturated fatty acid; MUFA, monounsaturated fatty acid; PUFA, polyunsaturated fatty acid.

General linear modeling procedures adjusted for age, gender, energy consumption, energy underreporting, community size, region, and maternal education level revealed a negative association between DII, KIDMED index score, and total dietary antioxidant capacity (Table 3). In contrast, dietary energy density (kcal/g) (*p* < 0.001) increased across quintiles of the DII. Including caloric beverages in the energy density calculation did not affect this association (*p* linear trend <0.001).

**Table 3.** General linear models of the association between diet quality and dietary inflammatory potential measured by the dietary inflammatory index (DII).

	Q1 (*n* = 578)	Q2 (*n* = 579)	Q3 (*n* = 577)	Q4 (*n* = 578)	Q5 (*n* = 577)	***p***^a^
Diet quality measures						
*Model 1^b^*						
Energy density (kcal/g)	0.94 (0.91; 0.97)	1.02 (0.99; 1.04)	1.07 (1.04; 1.09)	1.12 (1.09; 1.15)	1.20 (1.17; 1.23)	<0.001
TAC (mmol/L)	10.0 (9.33; 10.7)	9.40 (8.72; 10.0)	8.85 (8.17; 9.53)	9.27 (8.59; 9.96)	9.02 (8.33; 9.72)	0.068
KIDMED index (unit)	7.85 (7.67; 8.03)	7.33 (7.16; 7.51)	7.01 (6.86; 7.18)	6.91 (6.74; 7.08)	6.56 (6.39; 6.74)	<0.001
*Model 2^c^*						
Energy density (kcal/g)	0.95 (0.92; 0.98)	1.02 (1.00; 1.05)	1.07 (1.04; 1.09)	1.11 (1.09; 1.14)	1.19 (1.16; 1.22)	<0.001
TAC (mmol/L)	10.2 (9.51; 10.9)	9.40 (8.72; 10.0)	8.79 (8.11; 9.47)	9.21 (8.52; 9.90)	9.02 (8.31; 9.72)	0.030
KIDMED index (unit)	7.77 (7.59; 7.96)	7.33 (7.16; 7.50)	7.05 (6.88; 7.23)	6.93 (6.76; 7.10)	6.62 (6.44; 6.80)	<0.001
*Z *scores as diet quality measures						
*Model 1^b^*						
Energy density (kcal/g)	−0.37 (−0.45; −0.29)	−0.15 (−0.23; −0.07)	−0.01 (−0.08; 0.06)	0.14 (0.06; 0.22)	0.36 (0.29; 0.44)	<0.001
TAC (mmol/L)	0.07 (0.00; 0.15)	0.00 (−0.06; 0.08)	−0.05 (−0.12; 0.02)	−0.00 (−0.08; 0.07)	−0.03 (−0.11; 0.04)	0.068
KIDMED index (unit)	0.33 (0.25; 0.42)	0.09 (0.01; 0.17)	−0.05 (−0.13; 0.02)	−0.10 (−0.18; −0.24)	−0.26 (−0.35; −0.18)	<0.001
*Model 2^c^*						
Energy density (kcal/g)	−0.33 (−0.41; −0.25)	−0.13 (−0.21; −0.57)	−0.00 (−0.08; 0.07)	0.13 (0.05; 0.20)	0.35 (0.27; 0.43)	<0.001
TAC (mmol/L)	0.10 (0.02; 0.18)	0.00 (−0.06; 0.08)	−0.05 (−0.13; 0.01)	−0.01 (−0.08; 0.06)	−0.03 (−0.11; 0.04)	0.030
KIDMED index (unit)	0.30 (0.21; 0.38)	0.09 (0.01; 0.17)	−0.03(−0.11; 0.04)	−0.09 (−0.17; −0.01)	−0.24 (−0.32; −0.15)	<0.001

Data are shown as means and 95% confidence interval.

^a^*p* values were obtained by polynomial contrast.

^b^Model 1: adjusted for gender and age.

^c^Model 2: additionally adjusted for maternal education level, community size, region, energy, and energy underreporting. Energy was not included in the energy density analysis.

TAC, total antioxidant capacity.

We determined the degree of agreement between the three diet quality measures. Cohen’s *κ* showed poor strength of agreement between the KIDMED index and total dietary antioxidant capacity (*κ* = 0.011, *p* = 0.421), the KIDMED index and energy density (*κ* = −0.080, *p* = 0.001), and total dietary antioxidant capacity and energy density (*κ* = −0.083, *p* = 0.001).

No significant interaction between DII and energy overreporting and underreporting was found.

Sensitivity analysis was used to assess the robustness of our results under various scenarios. Multivariate linear regression analysis stratified by gender and age groups revealed no meaningful differences when stratified by gender and age [children (6–11 years), adolescents (12–17 years), and young adults (18–24 years)] (Supplementary Table 1).

## Discussion

In this cross-sectional study in Spanish youth, higher diet quality was associated with increased dietary anti-inflammatory potential in three conceptually different measures validated in the study population. The strength of agreement between the selected indices was poor and correlations between measures were poor to fair; the one indicator that scored highly on all three was greater dietary anti-inflammatory potential. This finding makes an important contribution to the debate about defining and measuring diet quality.

Diet plays a pivotal role in inflammatory status because foods and nutrients modulate inflammatory processes [1–3]. The DII was created to measure the inflammatory potential of diets [7], based on a systematic review of literature reporting the effect of diet on inflammation. Thus, the DII has been used to determine the inflammatory profile of various diets [16,17,30–32]. In 2009–2010 data from the US National Health and Nutrition Examination Survey, a macrobiotic diet showed a significantly higher anti-inflammatory potential compared with the average US diet [30]; similarly, the DII indicated that the macrobiotic diet had more anti-inflammatory potential than the average US diet (mean DII average score of −1.88 and 1.00, respectively). Assessment of the dietary inflammatory potential of fast food, Mediterranean, and macrobiotic diet plans revealed a strong pro-inflammatory potential for the fast food diet; the opposite was true for the Mediterranean and macrobiotic diets [31]. Furthermore, adopting a vegan, vegetarian, or pescovegetarian diet was linked to a higher dietary anti-inflammatory potential, compared to adopting a semi-vegetarian diet after 2 months of dietary intervention in overweight and obese study participants [16].

Nutritional epidemiology offers several approaches to assessing overall diet quality [18]. Numerous dietary indices have been developed in the attempt to measure overall diet quality [33–35]. A recently published study [17] investigated the association of three diet quality measures (Healthy Eating Index 2010, the Alternative Healthy Eating Index, and the Dietary Approaches to Stop Hypertension Index) with dietary inflammatory potential as measured by the DII. In young adults, they found an increase in the anti-inflammatory potential associated with higher scores, indicating a healthier diet, on all three instruments in young adults [17].

The present study used three conceptually different measures of diet quality – energy density, antioxidant capacity, and the KIDMED index – to test our hypothesis that higher diet quality is related to a more favorable dietary inflammatory potential in young people. These diet quality indicators capture different dietary dimensions and have been directly associated with healthy food consumption in the present population [22,36].

In both adult and child populations, diets with low energy density are characterized by a high consumption of healthy foods, such as vegetables and fruits, reflecting a high diet quality [36,37]. Total dietary antioxidant capacity is based on the rationale that a diet rich in foods with high antioxidant potential will help to counterattack the harmful effects of free radicals in body tissues [38]. Adherence to the Mediterranean diet, as measured by the KIDMED index, reflects *a priori* selection of healthy foods and food habits. Notably, our results showed a poor agreement between these indices, indicating different patterns of food consumption within a healthy diet according to the measure used. However, a common characteristic of all three indices was the decrease in dietary inflammatory potential as diet quality scores increased. The lower effect size of the total dietary antioxidant potential compared with energy density and KIDMED scores was somewhat surprising; the amount of pro-inflammatory nutrients in foods which also exhibit antioxidant properties, such as meat and sausages, offers one potential explanation.

Several studies have shown that energy density is positively associated with energy intake in adult and younger populations [36,39]. It was therefore somewhat surprising that energy density increased with the inflammatory potential of the diet, with a concomitant decrease in total energy intake, in the present study. However, this does not necessarily imply that individuals following a high energy density diet will also have higher energy intakes compared with those adhering to a low energy density diet. Numerous food combinations show differences in total energy content for a given unit of energy density. Further studies are needed to verify whether lower energy intake despite higher energy density is a characteristic of increased inflammatory dietary potential in other populations.

Consistent with results from a previous study by Serra-Majem and colleagues [21], KIDMED scores were higher, indicating a healthier diet, in large cities than in small cities; the DII scores showed the same trend. There is emerging evidence that large urbanized cities have better access to healthy and affordable food [40]. In contrast, access to affordable healthy food can be challenging in rural areas with lower population density [40].

A limitation of this study is the cross-sectional study design, which precludes drawing causal relationships. Furthermore, 24 h recalls have inherent limitations in individual dietary assessment, owing to daily variations in food intake. However, the sample size of 2599 participants was large enough to characterize group intakes. Energy misreporting is an inherent bias in dietary assessment data. Although no interaction was found between the DII and energy misreporting, we adjusted the final data analysis for this potential confounder in order to reduce measurement error. The study also has several important strengths, including a nationwide population-based sample and interviewer-guided completion of questionnaires.

In conclusion, good diet quality, indicated by higher KIDMED index scores, higher total dietary antioxidant capacity, and lower energy density, is positively associated with dietary anti-inflammatory potential as measured by the DII in the Spanish population aged 6–24 years.

## Supplementary Material

Supplementary_table_1.docx
