# Supplementary material for: Association of diet quality with dietary inflammatory potential in youth
Source: Food Nutr Res. 2017 Jun 7;61(1):1328961. doi: 10.1080/16546628.2017.1328961 (PMC5475290; doi:10.1080/16546628.2017.1328961)
Supplement: Supplementary_table_1.docx [file zfnr_a_1328961_sm9031.docx]

| **Supplementary table 1.** Sensitivity analysis of the association between Dietary inflammatory index (DII) and other dietary scores according to sex and age group.^a^ | | | |
| --- | --- | --- | --- |
|  | β | 95%CI | *P* |
| **Sex** |  |  |  |
| *Male (n = 1318)* |  |  |  |
| KIDMED index (unit) | -0.179 | -0.085, -0.047 | <0.001 |
| TAC (mmol/L) | 0.019 | -0.004, 0.008 | 0.498 |
| Energy density (kcal/g) | 0.239 | 0.421, 0.649 | <0.001 |
| *Female ( n=1571)* |  |  |  |
| KIDMED index (unit) | -0.185 | -0.078, -0.046 | <0.001 |
| TAC (mmol/L) | -0.075 | -0.009, -0.002 | 0.003 |
| Energy density (kcal/g) | 0.232 | 0.384, 0.584 | <0.001 |
| **Age** |  |  |  |
| *6 to 9 years (n=375)* |  |  |  |
| KIDMED index (unit) | -0.069 | -0.047, 0.009 | 0.189 |
| TAC (mmol/L) | -0.111 | -0.108, -0.006 | 0.030 |
| Energy density (kcal/g) | 0.282 | 0.372, 0.779 | <0.001 |
| *10 to 13 years (n=525)* |  |  |  |
| KIDMED index (unit) | -0.184 | -0.082, -0.030 | <0.001 |
| TAC (mmol/L) | -0.124 | -0.054, -0.010 | 0.004 |
| Energy density (kcal/g) | 0.324 | 0.412, 0.689 | <0.001 |
| **Table continues** |  |  |  |
|  |  |  |  |
| **Table continued** |  |  |  |
| *14 to 17 years (n=621)* |  |  |  |
| KIDMED index (unit) | -0.142 | -0.081, -0.027 | <0.001 |
| TAC (mmol/L) | -0.055 | -0.013, 0.002 | 0.126 |
| Energy density (kcal/g) | 0.179 | 0.248, 0.570 | <0.001 |
| *18 to 24 years (n=1367)* |  |  |  |
| KIDMED index (unit) | - 0.233 | - 0.099, -0.063 | <0.001 |
| TAC (mmol/L) | -0.047 | -0.007, 0.001 | 0.081 |
| Energy density (kcal/g) | 0.245 | 0.424, 0.656 | <0.001 |

^a^ All models are adjusted for community size, region, and maternal education status.

TAC: total dietary antioxidant capacity. KIDMED, there is no definition.
